# Toward Explainable Precision Nephrology: Machine Learning-Based Chronic Kidney Disease Prediction

**DOI:** 10.3390/biomedicines14071459

**Published:** 2026-06-27

**Authors:** Moiz Qureshi, Akm Azad, Hasnain Iftikhar, Paulo Canas Rodrigues

**Affiliations:** 1 Department of Statistics, University of Sindh, Jamshoro 76080, Pakistan; moiz@stat.qau.edu.pk; 2 Department of Statistics, Quaid-i-Azam University, Islamabad 45320, Pakistan; 3 Department of Mathematics and Statistics, Faculty of Science, Imam Mohammad Ibn Saud Islamic University (IMSIU), Riyadh 13318, Saudi Arabia; kazad@imamu.edu.sa; 4 Department of Statistics, University of Peshawar, Peshawar 25120, Pakistan; 5 Department of Statistics, Federal University of Bahia, Salvador 40170-110, Brazil; paulo.canas@ufba.br; 6 Department of Business Management, University of Pretoria, Pretoria 0002, South Africa

**Keywords:** chronic kidney disease, artificial intelligence, explainable AI, machine learning, precision medicine, clinical decision support, feature selection, SMOTE, personalized healthcare, sustainable development goals

## Abstract

**Background/Objectives:** Chronic kidney disease (CKD) is an incurable and progressive condition; if diagnosed at an early stage, it would significantly reduce the risk of complications and enhance the outcomes for the patient. **Methods:** In this study, a custom dataset of 380 instances and 20 clinical attributes was used to develop and evaluate the machine learning (ML) models for reliable CKD prediction and to enhance the interpretability using explainable artificial intelligence (XAI) techniques. Artificial neural networks, C5.0, CHAID, logistic regression, linear support vector machines (L1 and L2 regularization), k-nearest neighbors (KNN), random tree, and deep neural networks were implemented. Correlation-based methods, recursive feature elimination, and LASSO were used for feature selection. SMOTE and SMOTETomek resampling techniques were used to address class imbalance. Three experimental set-ups were considered: (i) using SMOTETomek, (ii) with and without SMOTE, and (iii) grouped features according to the strength of correlation (high, moderate, low). Accuracy, precision, recall, F1 Score, AUC, and Gini index were used to evaluate the model’s performance. The pipeline was implemented in Python using the scikit-learn and imbalanced-learn packages. **Results:** Using SHAP and LIME, model interpretability was improved, with the KNN classifier obtaining the highest accuracy of 94.74% without SMOTE, and the C5.0 model obtained the highest accuracy of 92.98% with SMOTE. In the feature-group experiments, the L1-regularized linear SVM achieved high accuracy (89.47%) with highly correlated features. In general, both resampling methods improved model robustness, and feature selection methods reduced the model’s dimensionality with little loss in performance. **Conclusions:** The ML framework proposed is promising in predicting CKD with high accuracy and interpretability with relevance. By combining feature selection with class balancing and explainable AI, the model’s performance improves, and its clinical trustworthiness is enhanced. The results indicate the potential in using ML-based decision support systems for early-stage CKD diagnosis and personalized healthcare.

## 1. Introduction

Chronic Kidney Disease (CKD) is an irreversible, progressive, and growing disease that moves towards the gradual decline in kidney function over time and is considered a major health burden in the world today [1,2]. CKD is a major cause of morbidity, mortality, and health care costs in low and middle-income countries where early diagnostic facilities are not widely available [3,4]. Many patients may not show any symptoms in the early stages and are diagnosed with the disease only when there is significant renal impairment, when dialysis or kidney transplantation are required [5,6,7]. Early diagnosis and prompt clinical management are therefore crucial for limiting disease progression, reducing complications, and improving patient survival outcomes [8,9,10]. In this context, AI-based healthcare technologies have proven effective in supporting precision medicine, personalized healthcare, and intelligent clinical decision-making systems [11,12,13,14].

With recent developments in electronic health records (EHRs), laboratory information systems (LIS), and healthcare digitization, it is now possible to gather large-scale, structured clinical data suitable for applying machine learning (ML) to predictive analytics [4,15,16,17]. Some clinical parameters, including serum creatinine, estimated glomerular filtration rate (eGFR), hemoglobin, albumin, blood pressure, diabetes status, and cardiovascular comorbidities, are commonly collected and can be well used for early prediction and prognosis assessment of CKD [18]. ML techniques are better at capturing nonlinear relationships and complex interactions among clinical variables, enabling better predictive performance and disease stratification compared with traditional statistical methods and clinician-based risk assessment [11,19,20,21]. Thus, AI-enhanced predictive models are being sought after for precision nephrology and personalized disease management.

Current studies on ML for the diagnosis of CKD are based on binary classification on a standard dataset, such as the UCI CKD dataset, which has 400 instances and 24 attributes [22,23,24]. This has been explored in a wide range of supervised learning algorithms such as the Random Forest (RF), Rotation Forest, Gradient Boosting, XGBoost, AdaBoost, LightGBM, logistic regression, support vector machines (SVM), k-nearest neighbors (KNN), multilayer perceptron (MLP), and deep neural networks (DNNs) [25,26,27]. Ensemble and tree-based models have achieved classification accuracy of 98–99 percent, and deep learning models have also shown high prediction accuracy of 98 percent [28]. For instance, the UCI dataset [23] has been used to test some of the above models, and they have been able to reach the near-perfect accuracy of 100%, while the other models that are based on DNN have been able to reach around 99.6–99.7% on the same data set [24]. Beyond binary diagnosis, a few studies have expanded ML frameworks to predict CKD stage and estimate long-term prognosis, including progression to end-stage kidney disease (ESKD) [3,4]. Two types of machine learning models: random survival forest and ensemble learning models, are found to be better in modeling nonlinear clinical risk factors associated with disease progression [18,29].

Despite the promising performance of ML models, clinical datasets are still prone to missing values, noise, and class imbalance, which can affect the reliability of predictions in generalization. Despite promising performance, clinical datasets are still affected by missing values, noise, and class imbalance, which may undermine the reliability of predictions for generalization [16,30,31]. Several preprocessing and optimization methods are thus proposed to enhance the predictive performance of the CKD models. Several methods such as missing value imputation via KNN and reconstruction using a generative adversarial network (GAN) are used to improve the quality of the data [12,30]. In the same way, synthetic oversampling techniques like Synthetic Minority Oversampling Technique (SMOTE), Borderline-SMOTE, Adaptive Synthetic Sampling (ADASYN), and SMOTETomek were used to tackle class imbalance issues [5,11,23]. Another commonly used method for feature scaling is to use gradient boosting methods and distance-based algorithms [32,33] to support these techniques. These pre-processing methods have been found to enhance the classification sensitivity, F1 score and AUC, especially in ensemble and margin-based learning methods [5,12].

Another key area of research in the context of AI powering CKD studies is feature selection and dimensionality reduction. Clinical datasets frequently present redundant or low-information attributes, so it is beneficial to develop a small set of clinically meaningful predictors to make the model more robust, easier to compute, and more clinically interpretable [11,23]. Feature selection techniques employed are correlation-based feature selection, chi-square analysis, mutual information, recursive feature elimination (RFE), LASSO regularization, Boruta, genetic algorithm, and principal component analysis (PCA) [5,32]. Previous work has shown that it is possible to drastically reduce the number of features while retaining the most informative clinical variables without loss of predictive performance, and even with an improvement [1]. Moreover, various ML frameworks have been optimized for feature engineering along with hyperparameter tuning, leading to the development of highly accurate and efficient CKD prediction systems [11,26].

While predictive performance is paramount, clinical use of ML systems must also be transparent and interpretable. However, many advanced ML and deep learning models are considered “black-box” models, which reduces clinicians’ trust in and adoption of these models in the healthcare environment. To overcome this challenge, there has been significant focus on explainable artificial intelligence (XAI) for CKD-related healthcare applications [26,30]. Some of the most popular XAI techniques for clinically important predictor identification and to explain individual model predictions at both global and patient-specific levels are Shapley Additive exPlanations (SHAP) and Local Interpretable Model-Agnostic Explanations (LIME) [24]. They identified serum CREA, eGFR, ALB, HGB, AGE and specific Gravity as the key risk factors for CKD in accordance with the published literature [26]. These interpretable AI systems help build clinicians’ trust, enhance decision transparency, and enable the seamless integration of AI-driven systems into clinical care.

In recent years, there has also been a focus on the use of AI-based CKD prediction tools via web-connected devices, medical Internet of Things (IoT) platforms, and intelligent clinical decision support systems (CDSS) [3,5]. These systems are designed to offer real-time risk assessment and tailored suggestions to clinicians and patients. However, a few challenges remain to be addressed, including a lack of external validation, limited generalizability across populations, privacy-preserving healthcare analytics, and a lack of interpretable frameworks tailored to real clinical workflows [12]. In addition, most previous works aim only to achieve the highest classification accuracy, without considering model transparency, feature correlation analysis, or comprehensive evaluation across various class-balancing conditions.

The present study aims to overcome these constraints by introducing a comprehensive, interpretable, and explainable AI framework for CKD prediction that incorporates feature selection, class balancing, and an integrated predictive pipeline. The current work examines the effect of feature grouping based on correlation strength and explores the performance of various ML classifiers in both balanced and imbalanced learning settings, in contrast to previous studies that primarily used all features without systematic interpretation. To identify predictors of clinical importance, recursive feature elimination, correlation-based analysis, and LASSO regularization are used, and to address class imbalance, SMOTE and SMOTETomek methods are applied. Furthermore, SHAP and LIME are integrated to make models more interpretable and provide clinically relevant explanations of prediction results. Experimental results confirm that the KNN classifier achieves the highest accuracy of 94.74% without SMOTE, while the C5.0 model achieves 92.98% accuracy with SMOTE-based resampling. The proposed framework thus supports the implementation of precise clinical decision support systems, with transparency, reliability, and the integration of artificial intelligence. The proposed framework thus helps build transparent and reliable AI-equipped clinical decision support systems for precision nephrology and the personalized management of CKD.

### Novelties and Contributions

The present study is based on the same clinical setting as in our previous study. The present study introduces new methodological and analytical developments that were not explored in previous work [2,13]. First, this study systematically evaluates correlation-based feature grouping by partitioning the predictors into high-, moderate-, and low-correlation subsets to assess each subset’s contribution to classification performance. This analysis was not done in the previous study. Second, the present work includes a more extensive comparative framework across various machine learning classifiers, feature selection, class imbalance handling strategies, and explainable artificial intelligence (XAI) techniques. Additionally, SHAP and LIME are used not only to interpret the model after training but also to provide clinically relevant information about the renal and urinary markers that contribute to CKD prediction. Fourth, new analyses—such as comparisons of classifiers within each correlation group, assessments of model interpretability, calibration evaluations, and error analyses have been added to enhance transparency and clinical relevance.

The present study is designed to explore how different levels of feature relevance and feature interdependence, model interpretability, and data-balancing strategies affect predictive performance and clinical explainability. Thus, the novelty of this work extends not only to the inclusion of more patients but also to the creation of a more complete and comprehensible analytical framework for predicting CKD and evaluating biomarkers. In addition, the machine learning and deep learning methods used in this study are widely applied, but the novelty lies in the systematic integration and evaluation of these techniques into a single predictive model for chronic kidney disease (CKD). This study can be summarized with respect to the following major contributions:

- To build a comprehensive CKD prediction framework, the data preprocessing, feature selection, class imbalance, machine learning classification, and explainable artificial intelligence (XAI) methods are integrated.
- Multiple feature selection methods, such as correlation analysis, Recursive Feature Elimination (RFE), and LASSO, are comparatively studied for the purpose of reducing the number of features to a small subset of the clinically relevant ones, with high prediction performance.
- The effect of class imbalance treatment methods (SMOTE and SMOTETomek) is systematically evaluated on the performance of various machine learning classifiers to examine how these methods affect the performance of predicting CKD.
- Two explainability methods (SHAP and LIME) are used, both at global and at local level to gain clinically explainable understanding of the factors that drive model predictions.
- The study shows that a relatively small number of informative clinical variables can perform competitive classification, which also facilitates the development of efficient and interpretable CKD screening tools.

Materials and methods are presented in Section 2, which describes the data, preprocessing, feature selection, and ML modeling procedures. The experimental results and interpretability analysis are discussed in Section 3. Section 4 compares the proposed framework with the state-of-the-art CKD prediction studies from the literature. Lastly, a section on key findings, limitations, and future research direction is presented at the end of the study in Section 5.

## 2. Materials and Methods

In this study, we present an interpretable machine learning (ML) system that predicts Chronic Kidney Disease (CKD) from structured clinical data at an early stage. The proposed framework is consolidated into a Python 3.12.4 (Python Software Foundation, Wilmington, DE, USA) pipeline using the libraries scikit-learn and imbalanced-learn, which encompass all the above-mentioned steps, including data preprocessing, feature selection, class imbalance handling, predictive modeling, and explainable artificial intelligence (XAI). The methodology aims to enhance the prediction accuracy and interpretability of AI-based clinical decision support systems and precision medicine.

### 2.1. Dataset Description and Preprocessing

The CKD dataset used in this study comprises 382 patient records described by 20 clinical attributes and a binary target variable indicating the presence or absence of chronic kidney disease (CKD). Of the 382 patients, 258 (67.5%) were diagnosed with CKD, while 124 (32.5%) were classified as non-CKD. The dataset includes both numerical and categorical variables representing demographic characteristics, laboratory measurements, and urinalysis findings.

This research work is based on a structured clinical record of patients with chronic kidney disease (CKD) in District Buner, KPK, Pakistan. The clinical variables are demographic data (age and sex) and laboratory urine analysis parameters (albumin, glucose, blood, pus cells, red blood cells, bacteria, and cellular casts). The structured clinical attributes were used as predictor variables to create and assess the machine learning models. Details of all variables are given in Table 1. The data used in this study is available from: https://www.researchgate.net/publication/372689997_Chronic_kidney_disease_patients_from_district_Buner_Khyber_Pakhtunkhwa_Pakistan (accessed on 25 May 2025). The dataset includes anonymized clinical information and can be used for research and academic purposes. The detection of Chronic Kidney Disease (CKD) is formulated as a binary classification problem, and supervised learning algorithms are employed to predict the disease status. Let $X=[x_{1},x_{2},\ldots ,x_{n}]$ be the set of predictor variables (with $x_{n}$ being the least significant value), and let Y∈{0,1} be the binary outcome variable, where Y=1 represents the presence of CKD and Y=0 represents the absence of CKD.

The data set contains both quantitative and qualitative clinical data. All variables, along with nominal attributes, are provided with a coding scheme in Table 1 of [2]. The categorical variables were coded for use by machine learning algorithms. Special attention was given to the nominal variables, which were transformed into numerical form via label encoding. Label encoding might be used to implicitly encode an ordinal relationship among categories, but it was used for computational efficiency and to make the labels compatible with the selected classifiers. To reduce potential bias, all encoded variables were evaluated with different machine learning models, and the results were consistent across all algorithms.

Prior to model development, the dataset was preprocessed to improve data quality and ensure compatibility with ML algorithms. Missing values in numerical variables were imputed using median imputation, which is robust against outliers and skewed distributions. The imputation process is mathematically expressed as

(1) $$x_{ij}=\mathrm{median}\left(x_{j}\right)$$

where $x_{ij}$ denotes the missing value of the *j*th feature for the *i*th observation.

To reduce scale variability among numerical features, standardization was performed using z-score normalization:

(2) $$z_{ij}=\frac{x_{ij}-\mu_{j}}{\sigma_{j}}$$

where $\mu_{j}$ and $\sigma_{j}$ represent the mean and standard deviation of the *j*th feature, respectively. Categorical variables were transformed using label encoding to facilitate numerical computation during model training.

### 2.2. Feature Selection and Dimensionality Reduction

Feature selection was performed to reduce dimensionality, improve model generalization, and identify clinically relevant predictors associated with CKD. Three complementary feature selection techniques were employed: correlation-based feature selection, Recursive Feature Elimination (RFE), and Least Absolute Shrinkage and Selection Operator (LASSO).

Correlation analysis was used to categorize features based on their associations with the target variable. RFE iteratively eliminated less informative variables based on classifier importance scores, whereas LASSO performed embedded feature selection through coefficient regularization. The LASSO optimization problem is defined as

(3) $$\min_{\beta }\left\{\sum_{i=1}^{n}{\left(y_{i}-x_{i}^{T}\beta \right)}^{2}+\lambda \sum_{j=1}^{p}\left|\beta_{j}\right|\right\}$$

where λ controls the degree of sparsity and $\beta_{j}$ denotes the regression coefficients.

These feature engineering strategies reduce redundant clinical attributes while preserving diagnostically significant information, thereby supporting efficient and interpretable healthcare analytics.

### 2.3. Class Imbalance Handling

Clinical datasets frequently exhibit class imbalance, which may bias predictive models toward the majority class [34]. To address this limitation, Synthetic Minority Oversampling Technique (SMOTE) and SMOTETomek were employed.

SMOTE generates synthetic minority-class samples using interpolation between nearest-neighbor observations:

(4) $$x_{\mathrm{new}}=x_{i}+\delta \left(x_{zi}-x_{i}\right)$$

where $x_{i}$ represents a minority-class sample, $x_{zi}$ is one of its nearest neighbors, and δ∈[0,1] is a random interpolation coefficient.

SMOTETomek combines synthetic oversampling with Tomek link undersampling to remove overlapping observations near class boundaries, thereby improving class separability and reducing classification bias.

All pre-processing and development of models were done only in the training data in each iteration of cross-validation to avoid the leakage of data and a biased assessment of the model performance. In particular, missing-value imputation, feature scaling, feature selection (correlation analysis, RFE, and LASSO), handling class balance (SMOTE and SMOTETomek), and hyperparameter tuning were applied only to the training folds. The validation fold was then transformed in a similar fashion, and the selected features were used, with no knowledge of the validation data during model training. This procedure accounted for the model’s ability to generalize to previously unseen data and produced performance estimates that reflected it.

### 2.4. Machine Learning Models

Multiple supervised ML algorithms were implemented and comparatively evaluated to identify the most reliable model for CKD prediction.

#### 2.4.1. Logistic Regression

Logistic Regression (LR) is a probabilistic linear classifier widely used in medical diagnosis because of its interpretability and computational efficiency. The model estimates the probability of CKD occurrence using the logistic sigmoid function:

(5) $$P\left(y=1|x\right)=\frac{1}{1+e^{-(\beta_{0}+x^{T}\beta )}}$$

where $\beta_{0}$ represents the intercept and β denotes the coefficient vector.

#### 2.4.2. Artificial Neural Network

Artificial Neural Networks (ANNs) are biologically inspired computational models composed of interconnected neurons arranged in layers [35]. In this study, a feed-forward ANN with a single hidden layer was employed to capture nonlinear relationships within high-dimensional clinical data. The network consists of one hidden layer with 32 neurons, selected based on empirical performance during cross-validation.

(6) $$h^{\left(l\right)}=\sigma \left(W^{\left(l\right)}h^{\left(l-1\right)}+b^{\left(l\right)}\right)$$

where $W^{\left(l\right)}$ and $b^{\left(l\right)}$ represent the weights and bias terms of the *l*-th layer, and σ(·) denotes the activation function applied at each layer.

#### 2.4.3. Support Vector Machine

Support Vector Machine (SVM) constructs an optimal separating hyperplane that maximizes the margin between classes. Linear SVM was employed due to its effectiveness for structured healthcare datasets [36]:

(7) $$f\left(x\right)=w^{T}x+b$$

The optimization objective is defined as

(8) $$\min_{w}\frac{1}{2}{∥w∥}^{2}+C\sum_{i=1}^{n}\xi_{i}$$

where *C* controls the trade-off between classification accuracy and margin maximization.

#### 2.4.4. K-Nearest Neighbors

K-Nearest Neighbors (KNN) is a non-parametric instance-based learning algorithm that assigns class labels according to the majority class among neighboring observations:

(9) $$\hat{y}=\mathrm{mode}\left\{y_{i}:x_{i}\in N_{k}\left(x\right)\right\}$$

where $N_{k}\left(x\right)$ denotes the set of *k* nearest neighbors. Cross-validation was performed on the training folds to find the best *k* for optimal classification performance.

#### 2.4.5. Decision Tree Models

Decision tree models, including C5.0 and CHAID, recursively partition the feature space into homogeneous subgroups using splitting criteria based on entropy or chi-square statistics. The Gini impurity index used for node splitting is defined as

(10) $$G=1-\sum_{k=1}^{K}p_{k}^{2}$$

where $p_{k}$ represents the probability of class membership.

#### 2.4.6. Random Tree

Random Tree classifiers introduce stochasticity in feature selection and splitting, thereby improving generalization and reducing overfitting. These models are particularly effective for handling complex nonlinear clinical relationships.

#### 2.4.7. Deep Neural Network

A fully connected Deep Neural Network (DNN) architecture was used to model the nonlinear relationship between the clinical variables and CKD status. The network structure comprised an input layer comprising the selected predictor variables, multiple hidden layers, and an output layer for binary classification [37]. Assume that the feature vector is *x*. The DNN is trained to learn the hierarchical representation, layer by layer, using nonlinear transformations:

(11) $$f\left(x\right)=\sigma_{L}\left(W_{L}\sigma_{L-1}\left(\cdots \sigma_{1}\left(W_{1}x+b_{1}\right)\right)+b_{L}\right),$$

Here, $W_{l}$ and $b_{l}$ are the weight matrix and bias vector for the *l*-th layer, respectively, and $\sigma_{l}\left(\cdot \right)$ is the activation function. In this study, a fully connected feed-forward DNN with three hidden layers of 64, 32, and 16 neurons, respectively, was used. The hidden layers were activated by the Rectified Linear Unit (ReLU), and the output layer was activated by the Sigmoid function to estimate the probability of CKD occurrence. A binary cross-entropy loss function was chosen, and the network parameters were optimized with the Adam optimizer at a learning rate of 0.001. The training was performed using dropout regularization (20% dropout rate) and early stopping to avoid overfitting. Using a maximum of 100 epochs and a batch size of 32, the model was trained. The hyperparameters were determined by cross-validation and hyperparameter tuning in the training folds.

### 2.5. Model Validation and Performance Evaluation

Stratified sampling was employed to split the data into training and testing subsets so as to maintain the class distribution in both sets. Randomized Search Cross-Validation (RandomizedSearchCV) was used in conjunction with five-fold cross-validation to enhance robustness and generalizability, further optimizing hyperparameters.

Several classification metrics, including accuracy, precision, recall, F1-score, Area Under the Receiver Operating Characteristic Curve (AUC), and Gini index, were used to evaluate the predictive performance of each model. These are complementary indicators of the model’s discrimination, sensitivity, and robustness in imbalanced learning. Table 2 summarizes the evaluation metrics used in this study.

### 2.6. Explainable Artificial Intelligence

To improve model transparency and support clinically interpretable predictions, Explainable Artificial Intelligence (XAI) techniques were integrated into the proposed framework. SHapley Additive exPlanations (SHAP) were used to quantify the contribution of individual features to prediction outcomes based on cooperative game theory:

(12) $$\phi_{i}=\sum_{S}\frac{|S|!(|F|-|S|-1)!}{|F|!}\left[f(S\cup \{i\})-f(S)\right]$$

where $\phi_{i}$ denotes the Shapley value of feature *i*.

In addition, Local Interpretable Model-Agnostic Explanations (LIME) were employed to generate local surrogate explanations around individual predictions:

(13) $$L\left(f,g,\pi_{x}\right)=\sum \pi_{x}\left(z_{i}\right){\left(f\left(z_{i}\right)-g\left(z_{i}\right)\right)}^{2}$$

where *g* represents the interpretable surrogate model and $\pi_{x}$ defines locality weighting around the target instance.

The integration of SHAP and LIME enhances the interpretability, transparency, and clinical trustworthiness of the proposed AI-driven CKD prediction framework, thereby supporting its applicability in precision medicine and intelligent clinical decision support systems.

## 3. Experimental Results

The proposed pipeline was implemented using Python libraries including scikit-learn, imbalanced-learn, SHAP, and LIME. The final selected features include {co, blood, red_cells, bpigment, cc, mt, bacteria, kb, u_clarity, gc, albumin, pus_cells, sugar}.

### 3.1. Performance with Combined Feature Selection

**Figure 1:**
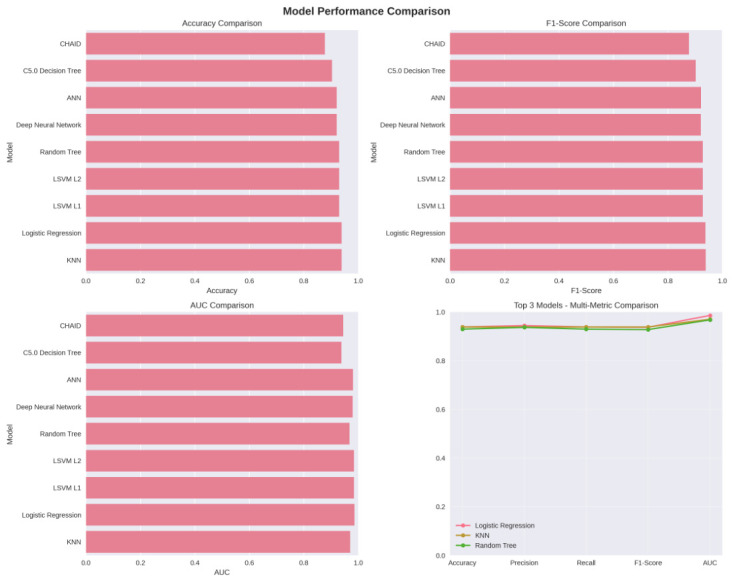
Comparison of model performances.

Figure 1 and Table 3 report the performance of classifiers with the chosen feature set. The findings indicate that all models achieve high prediction performance, with accuracies exceeding 87%. KNN was the most effective model, achieving the highest accuracy (94.74%), F1-score (94.76%), and AUC (0.9759). LSVM and Logistic Regression also showed good performance, with high AUC values (over 0.98), indicating strong discrimination. Conversely, CHAID demonstrated relatively poor performance in all metrics. Lastly, the findings indicate that instance-based and linear models can work effectively on the chosen feature space.

### 3.2. Impact of SMOTE

**Figure 2:**
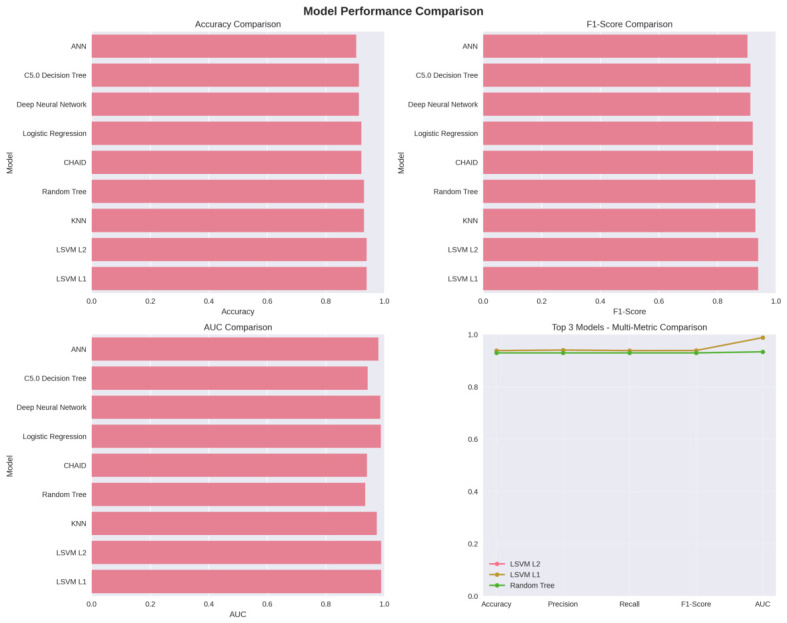
Performance of Classifiers with SMOTE.

Based on the multiple evaluation metrics like Accuracy, F1-score, and Area Under the Receiver Operating Characteristic Curve (AUC), a complete performance comparison of eight machine learning classifiers for chronic kidney disease (CKD) prediction is shown in Figure 2. The following models are evaluated: LSVM-L1, LSVM-L2, K-Nearest Neighbors (KNN), Random Tree, CHAID, Logistic Regression, Deep Neural Network (DNN), C5.0 Decision Tree and Artificial Neural Network (ANN). The results showed that the accuracy, F1-score, and AUC of all models were above 0.90, indicating strong discriminative power in detecting CKD cases. The best overall performance is achieved by the LSVM-L1 and LSVM-L2 models, followed by the Random Tree model, as they are the most stable and competitive across all metrics, implying a very strong ability to handle the nonlinear relationships among the clinical variables. Clinically, this excellent performance indicates that the selected laboratory and demographic biomarkers (albumin, blood in urine, pus cells, glucose, specific gravity) provide substantial diagnostic information for differentiating CKD from non-CKD cases. Overall, the models’ ability to be effective in distinguishing diseased and non-diseased cases at various classification thresholds, as evidenced by the high AUC values, is significant, especially in the context of clinical screening, where there is a need to balance sensitivity and specificity.

The F1-score comparison shows balanced performance between precision and recall, indicating that the models perform well and can be used to identify patients with CKD while minimizing false positives to avoid unnecessary clinical treatment. In addition, the ANN and C5.0 Decision Tree models achieve high accuracy, indicating that both deep learning and rule-based, tree-based approaches can discover underlying nonlinear relationships between renal pathology and clinical variables. Finally, the accuracy, precision, recall, F1 score and AUC of the top three models is compared with each other using multiple metrics, which again demonstrates the consistency of the performance across all the metrics, with the LSVM based models performing slightly better at generalization across all of the above. This indicates that structured clinical datasets, which are mixed categorical and numeric, such as those used in CKD classification, are a good indicator of the effectiveness of margin-based learning methods.

**Figure 3:**
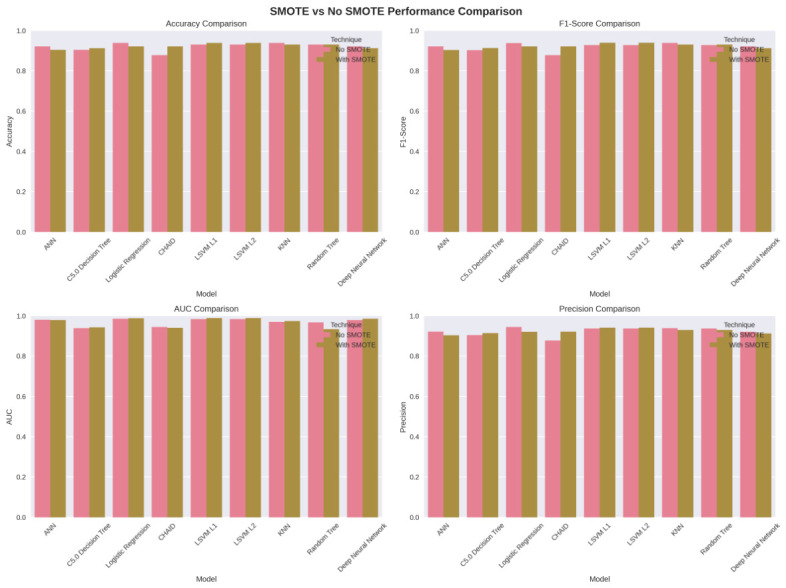
Performance of Classifiers comparison of with and without SMOTE.

Figure 3, together with Table 4 and Table 5 show a comparison of the performance of classifiers with and without the use of SMOTE for class imbalance. The findings showed that most classifiers achieved good baseline performance without SMOTE, indicating that the original data set contains sufficient discriminatory information for classifying chronic kidney disease (CKD). Specifically, K-Nearest Neighbors (KNN) and Deep Neural Network (DNN) have shown high predictive performance with an accuracy of around 94.74% when applied in the non-SMOTE setup. Logistic Regression also performs well, with a consistently high AUC of 0.9858, indicating good separation between the CKD and non-CKD classes. A minor increase in performance is seen for the models after applying SMOTE. Some classifiers show minor variations in accuracy, such as KNN and DNN, with a slight decrease to about 92.11%. This implies that synthetic oversampling may alter class distributions, thereby affecting distance-based and deep learning models that rely on the data’s distributional properties. Tree-based models are relatively unaffected by class imbalance handling techniques, such as C5.0 and CHAID. Overall, the data is moderately balanced, and the model’s performance is primarily due to feature separability rather than class balancing.

### 3.3. Correlation-Based Feature Analysis

**Figure 4:**
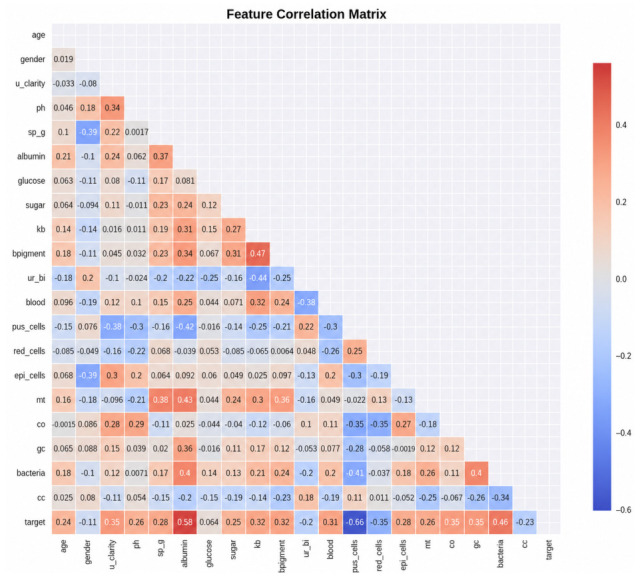
Correlation Matrix of All Considered Variables.

The statistical basis of the correlation-based grouping strategy employed in this study is given in the feature correlation matrix (Figure 4). The matrix shows the relationships between renal/urinary system biomarkers and the target variable (CKD status). The absolute correlation value of the variables with the largest value are more strongly associated with the outcome than those with the smaller value are. Albumin (r = 0.58), bacteria (r = 0.46), blood pigment (r = 0.32), blood (r = 0.31), and specific gravity (r = 0.28) showed relatively higher correlations with CKD status and were therefore considered more informative predictors.

Correlation groups are standardized to avoid inconsistencies in their definition. The features were organized by using an absolute correlation threshold: high correlation (|r|≥0.4), moderate correlation (0.3≤|r|<0.4), and low correlation (|r|<0.3). This corrected and consistent grouping criterion is now applied throughout the manuscript.

**Figure 5:**
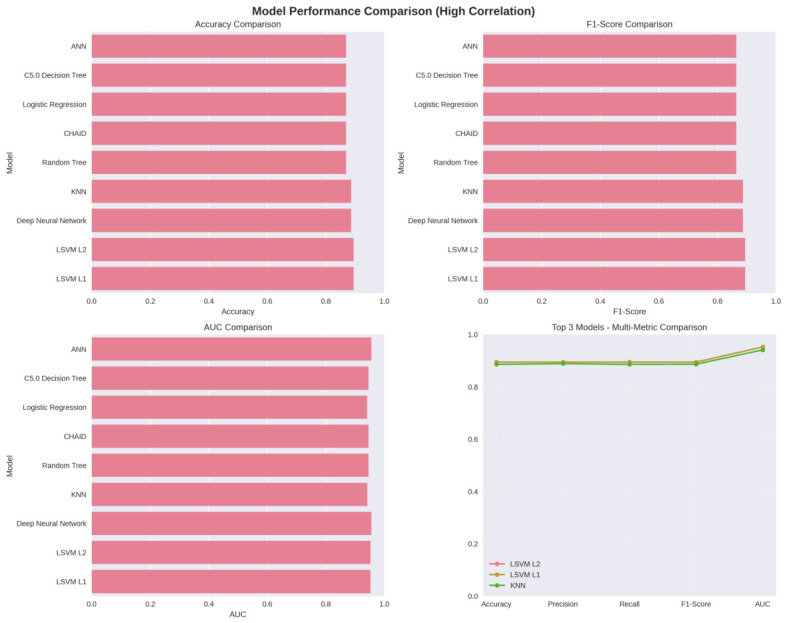
Comparative Performance of All Considered Predictive Models Using Highly Correlated Features.

**Figure 6:**
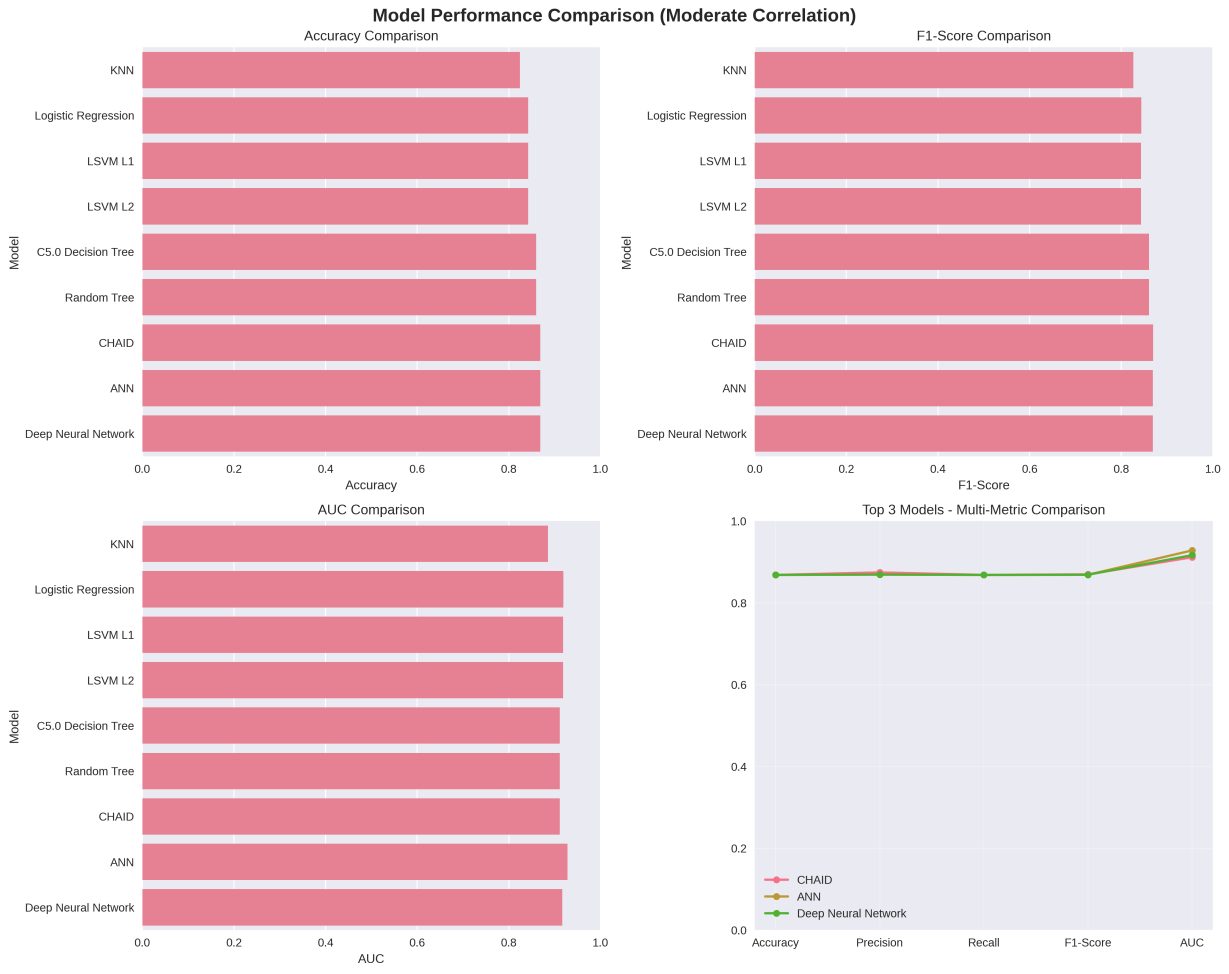
Comparative Performance of All Considered Predictive Models Using Moderate Correlated Features.

**Figure 7:**
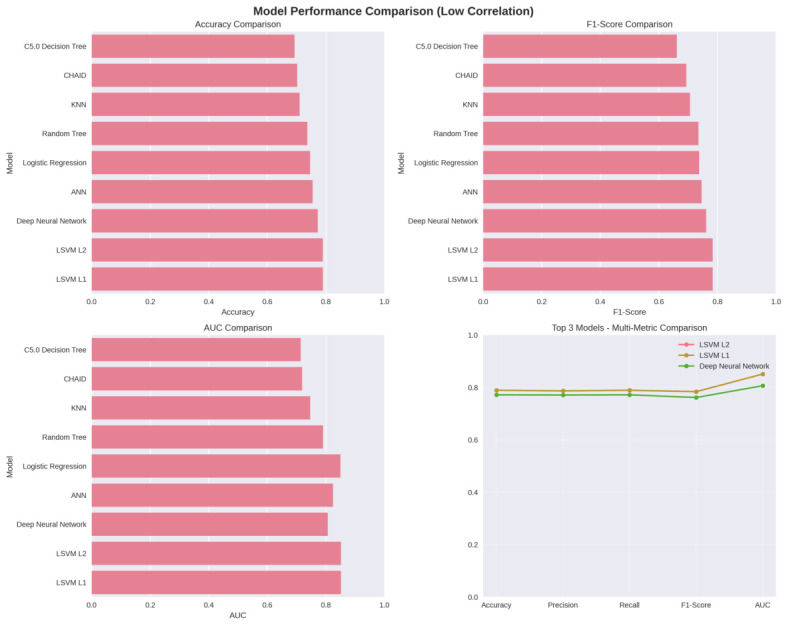
Comparative Performance of All Considered Predictive Models Using Low Correlated Features.

Figure 5, Figure 6 and Figure 7, present classifier performance across different correlation-based feature groups, while Table 6, Table 7 and Table 8 summarize the corresponding results. LSVM achieved the highest accuracy of 89.47% when trained on the high-correlation feature group (Table 6). This indicates that features with stronger statistical association with CKD contribute more predictive power to the classification models.

The moderate-correlation group’s performance was slightly below that of the other groups, with accuracy between 85% and 87%. This group showed CHAID performed well relative to other methods, indicating that tree-based models are better able to detect the complex interactions among moderately correlated variables.

The performance of the low-correlation feature group, on the other hand, was significantly degraded, with all the classifiers showing less than 80% accuracy. These variables have lower statistical correlations with the target variable and hence lower discriminative ability; this can cause noise in the learning process.

A comparative summary of the performance of each of the classifiers in each of the correlation groups is summarized in Table 9. The results clearly show that models trained on a highly correlated feature set always outperform models trained on a moderately or weakly correlated feature set. This is consistent with the hypothesis that clinically and statistically relevant biomarkers improve the predictive accuracy and robustness of models.

Each feature was correlated with the outcome variable, and the correlation strength of each feature with the outcome variable was calculated and used to rank the features for further analysis. On the basis of the corrected grouping strategy, the features were divided into high, moderate, and low correlation groups to study their contribution to the performance of the prediction of CKD.

The high-correlation group (|r|≥0.4) included variables such as *pus_cellsretain* (r = −0.6634), *albumin* (r = 0.5801), and *bacteria* (r = 0.4615). The strongest associations with CKD status were found in these features; thus, they contributed substantially to the predictive information. From a clinical point of view, albuminuria is a sign of glomerular damage, while the presence of pus cells and bacteria is an indicator of infection and inflammation, a frequent feature in patients with CKD.

The moderate-correlation group (0.3≤|r|<0.4) consisted of variables such as *u_clarity*, *kb*, *bpigment*, *blood*, *red_cells*, *co*, and *gc*. These variables provide supplementary information on renal dysfunction and metabolic imbalance, but have weaker predictive power than the high-correlation group.

The low-correlation group (|r|<0.3) included features such as *age*, *gender*, *ph*, *sp_g*, *glucose*, *sugar*, *ur_bi*, *epi_cells*, *mt*, and *cc*. These variables showed a weak linear association with CKD and contributed less to classification performance.

The grouping framework is corrected in order to be consistent across all experiments, and to overcome the previously mentioned lack of consistency in the definition of the high-correlation group. This strengthens the methodological transparency and interpretability of the feature selection strategy.

The results of the correlation-based grouping analysis show that renal and inflammatory markers that are strongly correlated are important to predict CKD. The findings also validate the clinical significance of proteinuria, infection, inflammation, and metabolic dysfunction in the progression of CKD. Therefore, choosing more informative correlated features will increase the accuracy, robustness, and interpretability of the model and will decrease noise from bad predictors.

### 3.4. Explainable AI Analysis

**Figure 8:**
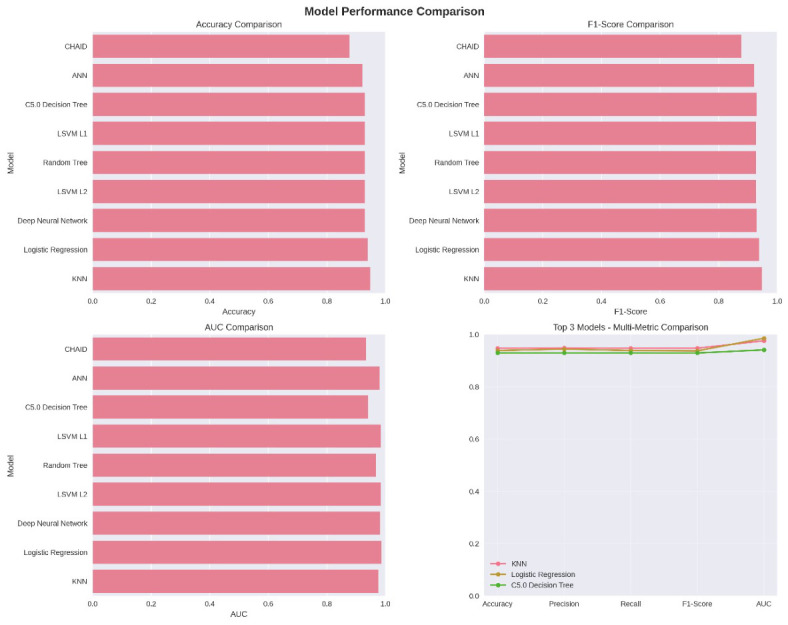
SHAP Model Performance Comparison.

The results of the overall classification presented above (Table 10 and Figure 8) are consistent with the results of the classification obtained from the above classification model, with the KNN model having the best classification overall. The three most important predictors in the SHAP analysis for classifying the disease as CKD were *albumin*, *pus_cells*, and *blood*. These findings are supported by the correlation analysis and the clinical literature on CKD progression, which identifies albuminuria, hematuria, urinary abnormalities, and inflammatory processes as key indicators of impaired renal function and disease progression. The major contribution of these variables also reflects the high predictive power of clinical biomarkers that are highly correlated and their significant impact on the model.

Moreover, the results of the SHAP in the model performance Table 10 show that some features associated with urinary abnormalities, inflammation and renal dysfunction play an important role in the model prediction. Urinary albumin has a strong association with kidney damage, indicative of glomerular dysfunction and, in particular, progressive loss of renal filtration capacity. The variable blood indicates the presence of blood in urine (hematuria) and the variable $pus_{c}ells$ indicates the presence of leukocytes in urine (which may suggest the presence of a urinary tract infection, or renal inflammation). The findings indicated that these urinary biomarkers play a key role in identifying chronic kidney disease (CKD), which is consistent with clinical knowledge of disease progression. These factors were important in the SHAP analysis and suggest that proteinuria, hematuria, and urinary sediment abnormalities are important clinical variables to consider in the classification process. Other clinic parameters, including urine clarity, urobilinogen, glucose, and sugar, also contribute to the prediction results, indicating an upset in metabolism and renal function. The joint analysis of SHAP and LIME demonstrates consistency between the global and local explainability approaches, highlighting clinically relevant patterns associated with CKD, including inflammation, impaired filtration, and urinary tract abnormalities.

### 3.5. Ablation Study

An ablation study was performed to evaluate the role of each component in the pipeline proposed. The results are given in Table 11.

The findings show that each of these components helps to improve the overall performance, and that feature selection and data balancing are the most significant components.

### 3.6. Model Interpretability Using SHAP and LIME

To achieve interpretability, SHAP and LIME methods were used to interpret the predictions of the model at the global and local levels, respectively. The importance of each feature in the final prediction was quantified using SHAP values, thereby identifying the most important clinical features, including albumin, pus cells, and bacteria.

To achieve local explanations for individual predictions, LIME was used to generate explanations of how values of specific features affect the classification of a particular patient instance for CKD. SHAP and LIME are used together to provide transparency in the ML models and improve clinical interpretability.

## 4. Comparison with CKD Prediction Studies

The experimental results showed that the proposed CKD prediction model is highly robust and effective across various evaluation scenarios. In particular, the KNN classifier outperformed other classifiers with an accuracy of 94.74 percent and better F1 score and AUC, indicating good classification performance. The proposed framework is based on efficient feature selection and systematic evaluation (rather than complex architectures), unlike many existing studies that rely heavily on complex architectures, thereby enhancing the stability and generalizability of the proposed framework. High predictive performance for detecting CKD with machine learning methods has been reported in several studies. As representative examples, the SVM and KNN models achieved accuracy greater than 98% on the UCI data set [38,39]. Similarly, logistic regression and ensemble models have been shown to achieve accuracies above 97 percent [40]. Deep neural networks have proven to be highly effective in deep learning, with near-perfect accuracy reported in [41]. Nevertheless, these findings are often obtained under controlled experimental conditions and may be overfit or lack external validity, as noted in hybrid modeling [42]. The work presented in our previous study [2,13] concentrated on the evaluation of the classification performance of the machine learning and ensemble classifiers in CKD detection, whereas the current study broadens the scope of the previous study in some important directions. In particular, this work examines correlation-based feature grouping, assesses how feature relevance affects classification results, adds an explanatory dimension to the results with SHAP or LIME analyses, and provides a more extensive evaluation of model robustness and clinical interpretability. Thus, the present work has also highlighted the added value of the larger dataset and the creation of a more transparent and clinically informative framework for the prediction of CKD.

By contrast, the proposed study achieves competitive performance and offers a simple, interpretable model. The most interesting finding is that the high-correlation feature group comprising only three variables achieved 89.47% accuracy with LSVM. This indicates the efficacy of the proposed feature selection approach and is consistent with the results of [43], which highlighted the importance of feature reduction for improving model efficiency. In contrast to prior work that uses large feature sets, this paper shows that a small, meaningful subset of features can achieve similar performance at lower computational cost.

The second valuable contribution of this work is the systematic analysis of methods for addressing class imbalance. Although SMOTE is common in the CKD prediction literature, including [44], the findings in this work suggest that SMOTE does not necessarily improve performance and may introduce noise, especially in instance-based models such as KNN. The latter observation suggests that special attention should be paid to data properties before implementing resampling methods, a point that has not been adequately considered in earlier studies. Moreover, explainable AI methods are integrated into the study, which is why it differs from most published literature. Although previous studies primarily focus on predictive accuracy, the proposed framework employs both SHAP and LIME to provide global and local interpretability. The detection of clinically meaningful characteristics, including albumin and pus cells, aligns with domain knowledge and is consistent with recent research on the significance of interpretable models in healthcare applications [45,46]. This makes the model more open and credible, crucial for real-world clinical use, and makes it easier to see the advantages of a patient-centered approach. In general, the proposed study is superior to current approaches in three aspects. It not only has high, stable predictive power but also does not require complex, overfitted models. Second, it provides a systematic analysis of the effects of feature relevance and class imbalance, providing more methodological information. Third, it employs explainable AI techniques to ensure interpretability and clinical relevance. All of these contributions lead to an effective, practicable, and strong tool for forecasting CKD, as proposed by the framework.

## 5. Conclusions

This study used a comprehensive machine learning methodology for Chronic Kidney Disease (CKD) involving feature selection, feature balancing, multi-class prediction and XAI. Based on the empirical results, the feature group with the highest correlation (three features) achieved the highest LSVM accuracy (89.47%), indicating that feature relevance is a crucial factor in predictive model accuracy. This observation highlights that they can significantly reduce the number of features in the set while maintaining high information about the problem, without reducing the model’s accuracy. The accuracy of KNN on the original dataset (94.74%) was higher than that of the other classifiers, and C5.0 was the most stable in terms of balancing under SMOTE (92.98%). Additionally, the interpretability of the SHAP global and local effects, combined with LIME, gave the validation of the most important biomarkers, such as albumin, pus cells, and blood-related ones, reinforcing the clinical significance of the selected biomarkers. Policy-wise, the proposed framework can facilitate the development of data-driven healthcare systems by enabling early and accurate identification of CKD using only a few cost-effective clinical characteristics. In conclusion, these findings suggest that AI-powered diagnostic tools can play a crucial role in addressing healthcare challenges within resource-constrained environments. This identification of a few highly informative features also helps to implement targeted screening programs and standard diagnostic procedures.

The findings from this study are promising but need to be considered in light of the following limitations. The data set was small and came from a single healthcare center in Khyber Pakhtunkhwa, Pakistan, which could reduce the generalizability of the results to other people. In addition, the dataset did not include key sociodemographic data that could affect the risk of developing CKD and the disease’s progression. However, the proposed machine learning framework has high potential for prediction and interpretation, but should not be used in routine clinical practice without further validation. The proposed approach should be further tested in future studies using larger-scale, multi-center, and geographically diverse datasets, along with external validation sets. Future clinical research is also required to evaluate the approach’s validity, applicability, and generalizability in clinical practice. Even with these constraints, the findings suggest that insights into CKD prediction and biomarker importance can be gained through a combination of feature selection, machine learning algorithms, and explainable artificial intelligence techniques. The proposed framework could serve as a basis for future studies to develop reliable, clinically validated decision-support tools for CKD screening and risk assessment.

### Limitations and Future Work

Although the proposed system has shown good performance, there are some drawbacks that must be recognized to better understand the results. The study has a relatively small sample size, which could limit the generalizability of the results. When using small datasets, model overfitting may occur, and the models may lack robustness in predicting their outcomes for future clinical populations. Second, a significant limitation is its lack of external validation. Evaluation of the proposed model was conducted only through internal validation, so the model’s performance on an external, independent set of data from different hospitals or populations has not yet been tested. Additional research is needed to include multi-center data to evaluate generalizability and clinical reliability. Third, although careful preprocessing was performed, there is a risk of implicit data leakage if it is carried out prior to cross-validation during the feature selection and resampling stages. In the future, it is desirable to have all preprocessing steps fully within the training folds, using a fully pipeline approach, to alleviate this concern. Fourth, the clinical information in the data set is limited, and it may not fully capture the complexity of the onset of chronic kidney disease. Other significant biochemical, imaging features and indicators of comorbidities were not available. Finally, the study is based on retrospective data analysis. The model needs to be validated in clinical settings before it can be deployed in the real world. For future work, it is recommended that prospective studies and clinical trials should be done to test the models in real-time diagnostic settings.

## Figures and Tables

**Table 1:** Description of Clinical Attributes Used in the CKD Dataset.

Attribute	Description
age	Age of the patient in years (numerical)
ph	Urine pH level (numerical)
sp_g	Specific gravity (numerical)
gender	Gender of the patient (categorical)
u_clarity	Urine clarity (categorical)
albumin	Albumin level in urine (categorical)
glucose	Glucose level in urine (categorical)
sugar	Sugar concentration (categorical)
kb	Ketone bodies (categorical)
bpigment	Bilirubin pigment (categorical)
ur_bi	Urobilinogen level (categorical)
blood	Presence of blood in urine (categorical)
pus_cells	Presence of pus cells (categorical)
red_cells	Red blood cells (categorical)
epi_cells	Epithelial cells (categorical)
mt	Mucus threads (categorical)
co	Calcium oxalate crystals (categorical)
gc	Granular casts (categorical)
bacteria	Presence of bacteria (categorical)
cc	Calcium carbonate (categorical)

**Table 2:** Performance Evaluation Metrics.

Metric	Mathematical Definition
Accuracy	$\frac{TP+TN}{TP+TN+FP+FN}$
Precision	$\frac{TP}{TP+FP}$
Recall (Sensitivity)	$\frac{TP}{TP+FN}$
F1-score	$\frac{2\times \mathrm{Precision}\times \mathrm{Recall}}{\mathrm{Precision}+\mathrm{Recall}}$
AUC	Area under the ROC curve
GINI	2×AUC−1

**Table 3:** Performance of Classifiers with Combined Feature Selection and SMOTETomek (Balancing Skipped).

Model	Accuracy	Precision	Recall	F1-Score	AUC	GINI
ANN	0.9211	0.9208	0.9211	0.9208	0.9792	0.9583
C5.0 Decision Tree	0.9298	0.9297	0.9298	0.9294	0.9410	0.8819
Logistic Regression	0.9386	0.9440	0.9386	0.9372	0.9858	0.9716
CHAID	0.8772	0.8765	0.8772	0.8765	0.9340	0.8681
LSVM L1	0.9298	0.9368	0.9298	0.9280	0.9841	0.9683
LSVM L2	0.9298	0.9368	0.9298	0.9280	0.9841	0.9683
KNN	0.9474	0.9485	0.9474	0.9476	0.9759	0.9517
Random Tree	0.9298	0.9368	0.9298	0.9280	0.9674	0.9349
Deep Neural Network	0.9298	0.9298	0.9298	0.9298	0.9812	0.9623

**Table 4:** Performance of Classifiers without SMOTE.

Model	Accuracy	Precision	Recall	F1-Score	AUC	GINI
ANN	0.9211	0.9211	0.9211	0.9204	0.9821	0.9643
C5.0 Decision Tree	0.9298	0.9297	0.9298	0.9294	0.9410	0.8819
Logistic Regression	0.9386	0.9440	0.9386	0.9372	0.9858	0.9716
CHAID	0.9035	0.9033	0.9035	0.9027	0.9497	0.8995
LSVM L1	0.9298	0.9368	0.9298	0.9280	0.9841	0.9683
LSVM L2	0.9298	0.9368	0.9298	0.9280	0.9841	0.9683
KNN	0.9474	0.9485	0.9474	0.9476	0.9858	0.9716
Random Tree	0.9298	0.9368	0.9298	0.9280	0.9674	0.9349
Deep Neural Network	0.9474	0.9474	0.9474	0.9471	0.9854	0.9709

**Table 5:** Performance of Classifiers with SMOTE.

Model	Accuracy	Precision	Recall	F1-Score	AUC	GINI
ANN	0.9211	0.9208	0.9211	0.9208	0.9805	0.9610
C5.0 Decision Tree	0.9298	0.9297	0.9298	0.9294	0.9400	0.8800
Logistic Regression	0.9211	0.9208	0.9211	0.9208	0.9884	0.9769
CHAID	0.9298	0.9297	0.9298	0.9294	0.9400	0.8800
LSVM L1	0.9211	0.9234	0.9211	0.9216	0.9897	0.9795
LSVM L2	0.9211	0.9234	0.9211	0.9216	0.9897	0.9795
KNN	0.9211	0.9216	0.9211	0.9212	0.9691	0.9382
Random Tree	0.9298	0.9297	0.9298	0.9294	0.9400	0.8800
Deep Neural Network	0.9211	0.9211	0.9211	0.9204	0.9831	0.9663

**Table 6:** Performance for High Correlation Features.

Model	Accuracy	Precision	Recall	F1-Score	AUC	GINI
ANN	0.8684	0.8729	0.8684	0.8644	0.9549	0.9097
C5.0 Decision Tree	0.8684	0.8729	0.8684	0.8644	0.9454	0.8909
Logistic Regression	0.8684	0.8729	0.8684	0.8644	0.9410	0.8819
CHAID	0.8684	0.8729	0.8684	0.8644	0.9454	0.8909
LSVM L1	0.8947	0.8947	0.8947	0.8947	0.9524	0.9048
LSVM L2	0.8947	0.8947	0.8947	0.8947	0.9524	0.9048
KNN	0.8860	0.8887	0.8860	0.8867	0.9411	0.8823
Random Tree	0.8684	0.8729	0.8684	0.8644	0.9454	0.8909
Deep Neural Network	0.8860	0.8887	0.8860	0.8867	0.9549	0.9097

**Table 7:** Performance for Moderate Correlation Features.

Model	Accuracy	Precision	Recall	F1-Score	AUC	GINI
ANN	0.8684	0.8692	0.8684	0.8687	0.9282	0.8565
C5.0 Decision Tree	0.8596	0.8615	0.8596	0.8603	0.9114	0.8228
Logistic Regression	0.8421	0.8505	0.8421	0.8439	0.9193	0.8386
CHAID	0.8684	0.8745	0.8684	0.8698	0.9115	0.8231
LSVM L1	0.8421	0.8469	0.8421	0.8434	0.9183	0.8366
LSVM L2	0.8421	0.8469	0.8421	0.8434	0.9183	0.8366
KNN	0.8246	0.8332	0.8246	0.8266	0.8857	0.7715
Random Tree	0.8596	0.8642	0.8596	0.8608	0.9112	0.8224
Deep Neural Network	0.8684	0.8692	0.8684	0.8687	0.9167	0.8333

**Table 8:** Performance for Low Correlation Features.

Model	Accuracy	Precision	Recall	F1-Score	AUC	GINI
ANN	0.7544	0.7500	0.7544	0.7458	0.8241	0.6481
C5.0 Decision Tree	0.6930	0.6870	0.6930	0.6614	0.7141	0.4282
Logistic Regression	0.7456	0.7402	0.7456	0.7378	0.8492	0.6984
CHAID	0.7018	0.6933	0.7018	0.6939	0.7188	0.4375
LSVM L1	0.7895	0.7870	0.7895	0.7839	0.8512	0.7024
LSVM L2	0.7895	0.7870	0.7895	0.7839	0.8512	0.7024
KNN	0.7105	0.7048	0.7105	0.7062	0.7464	0.4927
Random Tree	0.7368	0.7345	0.7368	0.7354	0.7898	0.5797
Deep Neural Network	0.7719	0.7712	0.7719	0.7617	0.8065	0.6131

**Table 9:** Correlation Group Summary.

Correlation Group	Num Features	Best Model	Best F1	Best Acc	Avg Acc	Avg F1	Avg AUC
High	3	LSVM L1	0.8947	0.8947	0.8782	0.8761	0.9481
Moderate	7	CHAID	0.8698	0.8684	0.8528	0.8540	0.9134
Low	10	LSVM L1	0.7839	0.7895	0.7437	0.7345	0.7946

**Table 10:** SHAP Model Performance Metrics.

Model	Accuracy	Precision	Recall	F1-Score	AUC	GINI
ANN	0.9211	0.9208	0.9211	0.9208	0.9792	0.9583
C5.0 Decision Tree	0.9298	0.9297	0.9298	0.9294	0.9410	0.8819
Logistic Regression	0.9386	0.9440	0.9386	0.9372	0.9858	0.9716
CHAID	0.8772	0.8765	0.8772	0.8765	0.9340	0.8681
LSVM L1	0.9298	0.9368	0.9298	0.9280	0.9841	0.9683
LSVM L2	0.9298	0.9368	0.9298	0.9280	0.9841	0.9683
KNN	0.9474	0.9485	0.9474	0.9476	0.9759	0.9517
Random Tree	0.9298	0.9368	0.9298	0.9280	0.9674	0.9349
Deep Neural Network	0.9298	0.9298	0.9298	0.9298	0.9812	0.9623

**Table 11:** Ablation study of proposed framework components.

Model Configuration	Accuracy (%)
Full Model (All components)	89.47
Without SMOTE/SMOTETomek	86.10
Without Feature Selection (RFE/LASSO)	85.32
Without Correlation-Based Grouping	84.75
Without SHAP/LIME-guided validation	88.12

## Data Availability

The data used in this study are available at https://www.researchgate.net/publication/372689997_Chronic_kidney_disease_patients_from_district_Buner_Khyber_Pakhtunkhwa_Pakistan (accessed on 25 May 2025).
